# Towards Inhibition of Vif-APOBEC3G Interaction: Which Protein to Target?

**DOI:** 10.1155/2010/649315

**Published:** 2010-09-21

**Authors:** Iris Cadima-Couto, Joao Goncalves

**Affiliations:** URIA-Centro Patogénese Molecular and Instituto de Medicina Molecular, Faculdade de Farmácia da Universidade Lisboa, Avenue Das Forcas Armadas, 1649-019 Lisboa, Portugal

## Abstract

APOBEC proteins appeared in the cellular battle against HIV-1 as part of intrinsic cellular immunity. The antiretroviral activity of some of these proteins is overtaken by the action of HIV-1 Viral Infectivity Factor (Vif) protein. Since the discovery of APOBEC3G (A3G) as an antiviral factor, many advances have been made to understand its mechanism of action in the cell and how Vif acts in order to counteract its activity. The mainstream concept is that Vif overcomes the innate antiviral activity of A3G by direct protein binding and promoting its degradation via the cellular ubiquitin/proteasomal pathway. Vif may also inhibit A3G through mechanisms independent of proteasomal degradation. Binding of Vif to A3G is essential for its degradation since disruption of this interaction is predicted to stimulate intracellular antiviral immunity. In this paper we will discuss the different binding partners between both proteins as one of the major challenges for the development of new antiviral drugs.

## 1. Introduction

Vif is a 23-kDa cytoplasmic protein that is expressed from a partially spliced mRNA in Rev-dependent manner during the late phase of HIV-1 replication. The human immunodeficiency virus type 1 (HIV-1) Vif protein is essential for virus replication in primary lymphoid and myeloid cells, but is dispensable for efficient replication in several transformed T-cell lines as well as in nonlymphoid cell lines such as HeLa and 293T [1–3]. Cells that are unable to support the replication of Vif-defective HIV-1 (HIV-1∆Vif) have been termed “nonpermissive,” while cells that can sustain HIV-1∆Vif replication are termed “permissive.” 

These findings were made over 15 years ago but the molecular mechanisms underlying this cell-specific difference were maintained a mystery until 1998. The observation that heterokaryons formed by fusion of nonpermissive and permissive cells exhibit the nonpermissive phenotype [4, 5] led to the hypothesis that nonpermissive cells express an inhibitor of HIV-1 replication that is blocked by the viral Vif protein. Later, in 2002, Sheehy et al. reported the identification of the apolipoprotein B mRNA-editing enzyme-catalytic polypeptide-like 3G (APOBEC3G) as the HIV-1 replication inhibitor [6]. 

A3G is located in the cytoplasm of the cell and has been shown to be an exclusive DNA mutator [7]. A3G exists either as enzymatically active low-molecular-mass (LMM) forms consistent with enzyme monomers or dimers, or as an enzymatically inactive high-molecular-mass (HMM) ribonucleoprotein complex larger than 2 MDa [8]. LMM A3G is found in resting CD4+ T cells of peripheral blood and macrophages where it may act as a powerful antiviral restriction factor for HIV-1 [8]. Conversely, resting CD4+ T cells in lymphoid tissues are permissive to HIV-1 infection as A3G is expressed predominantly in HMM complexes due to the lymphoid microenvironment [9]. It was reported that in lymphoid tissues, cytokines such as IL-2 and IL-5 are responsible for the stimulation of HMM complexes, which in turn may confer the permissive phenotype for HIV-1 infection [9]. Nonetheless, only one research group have provided data to support a role for APOBEC3G in restriction of HIV-1 in quiescent CD4+ T cells. Therefore, to date the primary mechanism of HIV-1 restriction in quiescent CD4+ T cells remains to be elucidated.

## 2. The APOBEC3 Family

A3G belongs to a family of polynucleotide cytidine deaminases (CDAs), whose members include seven family members, named APOBEC3A to H (A3A–H). All of these genes are clustered on chromosome 22 [10, 11]. During mammalian evolution APOBEC3 (A3) family members have evolved from a single gene in mice, located on chromosome 15, to eight genes (A3A–H) in primates [10, 11]. Interestingly, expansion of the A3 gene cluster contrasts with the decline in retrotransposition activity in primates [10–12]. This observation raises the possibility that APOBEC3 genes may have evolved to prevent genomic instability caused by endogenous retroelements [13]. 

All members of the A3 family contain one (A3A, A3C, A3H) or two (A3B, A3DE, A3F, A3G) copies of a conserved zinc-binding motif, His/Cys-X-Glu-X _23-28_-Pro-Cys-X_2-4_-Cys, that catalyzes the hydrolytic deamination at the C4 position of 2′-deoxycytidine, resulting in a 2′-deoxyuridine [6, 10, 11, 14–16]. These proteins also contain a key glutamate required for proton shuttling during catalysis and two key aromatic residues involved in RNA substrate binding [16–22].

Proteins of the A3 family protect cells against the invasion of a variety of viruses [6, 23–25]. Of these, A3G and A3F are the most extensively studied due to their strong activity against HIV-1 [26]. Like A3G and A3F, A3B and A3DE contain two CDAs and have been shown to display moderate antiviral activity [23, 27–29]. Despite having only one CDA domain A3A, A3C, and A3H also impair HIV-1 infection [30–33].

The interplay between HIV-1 proteins and host restriction factors, such as A3G and A3F are potential targets for the development of new antiviral drugs. Inhibition of this interplay could allow the host innate defences to control viral replication. The interaction between Vif and A3G proteins has been extensively studied in the last decade and several regions and amino acid residues have been described as involved in the interaction between these two proteins. 

In this paper we will briefly describe the current knowledge on the different binding strategies between Vif and A3G, and discuss these mechanisms with the purpose of developing new antiviral drugs.

## 3. APOBEC3 Restriction of HIV-1 Infection

Expression of A3 proteins in HIV-1 infected cells can lead to their encapsidation into progeny virions through recruitment to viral or transposon capsid structures, probably involving Gag proteins and viral RNA [29, 34–37]. Deaminases will be delivered to the target cell where they will deaminate cytidines to uridines during the synthesis of the minus-strand viral cDNA [38, 39]. Consequently, during the synthesis of the plus-strand DNA, adenosines are incorporated instead of the original guanines resulting in G-to-A substitution. This process of deamination that will result in the loss of genetic integrity and protein function is commonly referred as hypermutation [40–42]. However, there is increasing evidence that A3G is able to restrict HIV-1 infection in the absence of deaminase activity [8, 43–45]. 

A study carried by Newman et al. showed that certain amino acid substitutions in the C-terminal cytidine deaminase “core” domain of A3G (APOBEC3G has two such domains) originated mutant proteins that were unable to mutate DNA, yet maintained the antiviral activity [44]. However, cytidine deaminase (CDA) independent effects cannot be observed when the mutant proteins are expressed at physiological levels [46], making these nonenzymatic A3G effects somewhat controversial. 

On the other hand, when unstimulated CD4+ T cells (where A3G is expressed as an active LMM form) were treated with A3G-specific small interfering RNAs (siRNA), the early replication block encountered by HIV-1 was greatly relieved [6]. When HIV-1 reverse transcripts in resting primary CD4 T cells were examined for evidence of A3G-induced dG → dA hypermutations, only 8% of the transcripts contained such mutations suggesting an antiretroviral activity independent of deoxycytidine deaminase activity [8]. 

Additional studies indicated that initiation of HIV reverse transcription and/or processivity of reverse transcriptase (RT) could be inhibited by A3G [47–52]. Sequence analyses of 2-LTR circle junctions from unintegrated DNA synthesized in the presence of A3G showed that the U5 end DNA occasionally had additional six RNA bases derived from the 3′-terminus of tRNALys3 [53]. These results suggest that A3G causes a defect during tRNA removal that limits plus-strand transfer and consequently affect viral DNA ends that will not be able to efficiently integrate into the host cell genomic DNA [53]. The process of successive inhibition of reverse transcription mechanisms has a cumulative effect and could explain end result of reducing viral integration. However, the decrease in plus-strand DNA transfer may not explain all the effects on viral cDNA synthesis by A3G [54]. Indeed, A3G could be coimmunoprecipitated with NC and integrase (IN) in HIV-1 Vif-positive viruses [55]. In addition, A3F co-immunoprecipitation with virion-associated integrase (IN) was also observed [55]. Nevertheless, GST-pull down assays do not show binding between A3G and RT, suggesting that interactions between A3G and viral proteins may may inhibit the process of reverse transcription [49].

## 4. APOBEC3 Restriction by Vif

**Figure 1:**
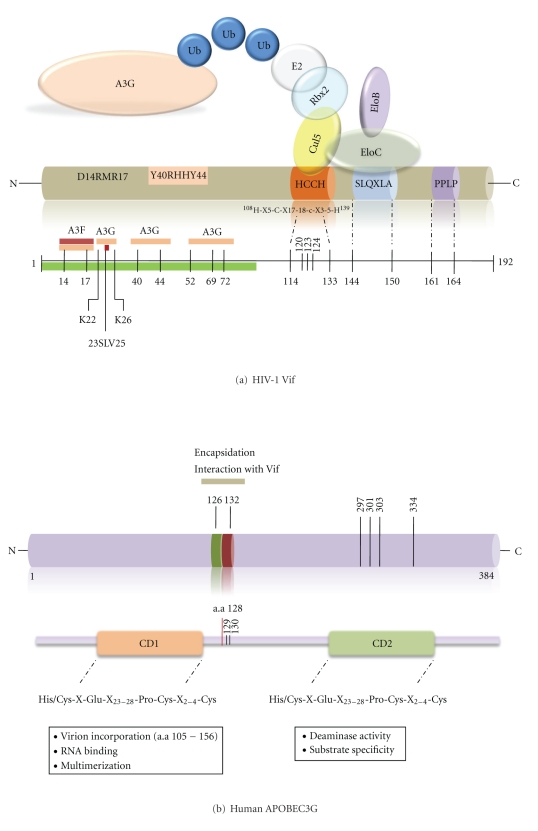
Schematic representation of Vif and A3G domains involved in the interaction of both proteins. (a) Vif binds A3G through specific residues located in the N-terminal region. Amino acids in Vif that are involved in the interaction with A3G are shown in pink. C-terminal Vif domains involved in targeting A3G for proteasomal degradation are shown in orange (zinc binding HCCH domain), and light blue (SLQXLA). The multimerization domain is purple. (b) The catalytic domains (CD1 and CD2) and Vif-binding regions of A3G protein are represented. Amino acids 126–132 are involved in A3G encapsidation and interaction with Vif and are represented in green and red.

To overcome the antiviral effect of APOBEC3 proteins, in particular A3G and A3F, HIV-1 encode the Vif protein. The mode of action by which Vif counteracts A3G and A3F-mediated antiviral activity has been extensively studied. Vif neutralizes the antiviral activity of A3G and A3F by forming a RING-finger E3 ubiquitin complex with Elongin B (EloB) and C (EloC), Cullin 5 (Cul5) and Ring-box protein 2 (Rbx2) (Figure 1(a)). By bringing A3G into contact with the RING-finger E3 ubiquitin complex, Vif promotes A3G polyubiquitination and its degradation in the 26S proteasome [56–63]. A more a recent report suggested that A3G needs Vif polyubiquitination to be degraded rather than its own polyubiquitination, [64], but this is still a matter of debate. Moreover, Vif has also been reported to directly block A3G encapsidation [57, 65, 66], reduce A3G translation [60, 65], and directly inhibit the catalytic activity of A3G [45]. It is still unknown whether all these mechanisms must operate in concert to inhibit A3G action. However, independently on the mode of action, the ultimate goal of Vif is to prevent A3G encapsidation into budding HIV-1 virions.

## 5. The Vif-A3G Interaction

Recent advances on the biological role of HIV-1 Vif and A3 proteins, together with progress in deciphering how Vif counteracts A3G and A3F opened new opportunities to develop anti-HIV drugs. However, understanding the mode of action of Vif and A3G alone can provide a number of attractive targets for drug development since A3G displays the most potent activity against HIV-1. 

Disruption of Vif-A3G interaction is predicted to rescue A3G expression and virion packaging, consequently stimulating intracellular antiviral activity. Similarly, pharmacologic studies to suppress A3G proteasome-mediated degradation have been shown to enhance A3G half life and consequently inhibit HIV-1 infection [59]. In order to facilitate the rational design of inhibitors for Vif-A3G interaction, experimental assays have been devised to define features of Vif that are involved in the interaction with A3G, and vice versa.

The N-terminal region of HIV-1 Vif is important for binding and neutralization of A3G and A3F and also contributes to species-specific recognition [58, 67–70]. In the C-terminal region of Vif, the highly conserved cysteine residues at positions 114 and 133 and the S^144^LQXLA^149^ motif (Figure 1(a)) are required for Vif function and HIV-1 replication [71, 72]_._ Vif associates with the Cul5-EloB-EloC complex by directly binding to EloC via a BC box motif at positions 144 to 150 and to Cul5 via hydrophobic residues at positions 120, 123, and 124 within a zinc-binding region formed by the highly conserved HCCH motif (Figure 1(a)) [73, 74]. The SLQXLA motif is essential for targeting A3G for proteasomal degradation. Substitution of the SLQ portion of the SLQXLA motif has been reported to be sufficient to prevent A3G degradation [57, 59, 75]. The zinc binding-motif HCCH is also involved in A3G degradation and necessary for the specificity of Vif-Cul5 interaction [76, 77]_._

Several groups have shown that Vif-induced degradation of A3G requires the physical interaction of the two proteins and that a single amino acid change in A3G at residue 128 was sufficient to abolish this interaction [78–80]. This assumption led to the conclusion that the Vif-A3G interaction is species-specific and is determined by aspartic acid at position 128 in A3G and lysine in African Green Monkey (AGM) [78–81]. Substitution of human A3G D128 by K128, found in AGM A3G, results in a mutant (D128K-A3G) protein that is resistant to the effect of Vif. This data can be explained either because the mutant protein is no longer able to interact with Vif or due to inhibition of subsequent downstream steps [68, 78, 79, 81]. 

Experiments using alanine-scanning and multiple synonym substitutions on A3G residues confirmed the central role played by the aspartic acid at position 128 and showed the crucial role of proline-129 and aspartic acid-130, as important contributory residues (Figure 1(b)) [82]. 

Specifically, resistance to Vif induced degradation was conferred by mutating the aspartic acid residue at position 128 or 130 to the positively charged residue lysine, indicating that the interaction between Vif and A3G is largely determined by electrostatic interactions involving these residues [82]_. _However, residue 128 has been shown to be more sensitive to amino acid alterations than 130, suggesting that amino acid D128 may play a more prominent role in A3G interaction with Vif [82]. 

Substitution of proline in residue 129 of A3G to alanine or glycine displayed a strong Vif-resistant phenotype indicating that a specific structural interaction is also required for an efficient inhibition of A3G by Vif [82]. In addition to electrostatic determinants, one study reported that A3G residues 54–124 were sufficient to coimmunoprecipitate Vif, suggesting the role of additional interacting domains between Vif and A3G [56]. Another study reported that amino acids 105–156 of A3G were sufficient for its interaction with Vif, and amino acids 157–245 were required for its degradation [83]. Recently, analysis of A3G chimeras identified amino acids 126–132 as critical determinants involved in Vif interaction (Figure 1(b)) [84]. Finally, by using model-guided mutagenesis, four Lys residues in the CDA of A3G (Lys-297, 301, 303, and 334) were recently identified as required for Vif-mediated polyubiquitination and degradation (Figure 1(b)) [85]. 

Asparagine at position 128 of A3G was shown to interact with amino acids 15 or 17 of Vif, and mutations in the D^14^RMR^17^ conserved region of Vif can also alter its species-specific effect [68]. Alteration of DRMR region to SERQ or SEMQ, which are present in SIV_AGM_ Vif, promotes the interaction of AGM A3G, rhesus (Rh) A3G, and D128K-A3G with HIV-1 Vif [68]. The loss of species restriction is probably caused by an overall increase in the negative charge of amino acids in the 14–17 region of HIV-1 Vif, which promotes effective interaction with the positive charge of lysine present at residue 128 in AGM A3G and Rh A3G. In addition, the DRMR region was also shown to be critical for the binding strength between A3G and Vif [68] although, additional interaction motifs were required for stabilization of this interaction (Figure 1(a)) [86]. 

By performing an extensive mutational analysis of Vif, Russell and Pathak identified the new motif Y^40^RHHY^44^, that was shown to be involved in binding to A3G (amino acids 126–132) (Figure 1(a)) [86]. Vif Y^40^RHHY^44^ motif was considered a critical domain for binding to A3G while the D^14^RMR^17^ domain could be involved in a secondary step involving A3G degradation [86]. 

Nonetheless, other amino acids in Vif may also contribute to A3G binding. Mehle et al. demonstrated that amino acids 40 to 71 in the N-terminus of Vif contain a nonlinear binding site for A3G and that His 42/43 are important for Vif-A3G binding and Vif-mediated degradation of A3G *in vivo* [87]. Another region of Vif comprising amino acids 52 to 72 was identified as responsible for Vif-mediated degradation and virion exclusion of A3G (Figure 1(a)) [88].

Recently, a highly conserved ^69^YXXL^72^ motif in Vif was shown to mediate the binding to human A3G and its subsequent degradation (Figure 1(a)) [88–90]. Pery et al. showed that this motif was critical for *in vitro* direct binding of recombinant GST-Vif (1–94 a.a) to A3G and by FRET assay [89]. Additionally, Vif residues 22–26 and Y30 were also involved in the interaction with APOBEC3 proteins [91, 92]. In particular, Vif K22 and K26 were shown to be important for degradation of A3G. Additionally, residue Y30 was involved in the interaction with both A3G and A3F raising the hypothesis that alteration of Tyr in position 30 may affect the conformational stability of Vif. Although alanine-scanning of ^23^SLV^25^ region did not reduce the ability of Vif to bind A3G and/or A3F, the antiviral effect was abolished [91, 92], reinforcing the idea that Vif binding to A3G does not necessarily lead to its degradation. 

Vif-mediated degradation of A3G is regulated by phosphorylation of Vif and A3G at Ser144 and Thr32, respectively [61, 93]. It was recently documented that phosphorylation of A3G by protein kinase A (PKA) reduces its binding to Vif affecting subsequent ubiquitination and degradation [93]. This finding indicates that phosphorylation events may also play an important role in the interaction between Vif and APOBEC3 proteins. 

The central hydrophilic domain, E^88^WRKKR^93^, and the proline-rich P^161^PLP^164^ domain (Figure 1(a)) of Vif have been implicated in enhancing its steady-state level and in binding to tyrosine kinases, respectively [94, 95]. It is conceivable that the E^88^WRKKR^93^ motif is involved in maintaining sufficient intracellular levels of Vif necessary for A3G inhibition. Mutations in the PPLP motif of Vif were shown to reduce the infectivity of virions produced in T cells and inhibit Vif-Vif interaction *in vitro* [96]. It has been suggested that multimerization of Vif may be necessary for A3G binding and PPLP region may be essential for this behaviour [97]. In addition, this region has also been identified as part of a novel “SOCS-box” motif implicated in binding to EloC [61, 62] and involved in the interaction of Vif with the cellular Hck tyrosine kinase [98, 99]. Recently, Donahue et al. demonstrated that mutations in PPLP motif impaired the ability of Vif binding to A3G, but did not affect EloC and Cullin5 binding [100].

Other additional studies have identified regions in Vif protein that are responsible for A3G and A3F inhibition but are not located in the SLQXLA and HCCH motifs. As an example, Simon et al. reported that any single amino acid substitutions in Vif sequences isolated from HIV-1 infected patients were sufficient to prevent A3G neutralization [67]_. _Moreover, a subset of mutants has been reported to be functional against A3G but not A3F and vice versa [86].

## 6. Conclusion Remarks

To this date, the most successful HIV-1 antiviral drugs in the market are those that target the HIV enzymes reverse transcriptase (RT) and protease (PR). Nevertheless, other strategies have proven to be highly effective such as integrase inhibitors [101–103], and entry inhibitors like T20 and Maraviroc [104]. 

As reviewed above, recent advances in the study of the biological and biochemical role of Vif and A3G, together with progress in deciphering how Vif counteracts A3G, opened new opportunities to develop novel anti-HIV drugs. 

Blocking the binding of Vif to A3G *in vivo* is certainly one of the most obvious therapeutic strategies. Several authors reported that Vif may function at multiple levels to prevent incorporation of A3G into viral particles [45, 60, 61, 105]. Therefore, preventing the binding of Vif to A3G may have two outcomes: (1) inhibition of A3G proteasomal degradation and, (2) increasing in the amount of A3G at viral assembly locations, resulting in a higher level of A3G incorporation into virions. However, care must be taken when attempting to increase A3G intracellular levels as A3G mRNA is highly expressed in some tumour cells [106], and this may potentially induce tumour formation. 

The amount of intracellular Vif can be reduced by degradation in the proteasome due to direct interaction with the SCF complex. Consequently, disruption of Vif-A3G interaction could prevent the proteolytic degradation of A3G and consequently increase the intracellular levels of Vif by impeding its destruction. Whether increasing amounts of Vif may rescue viral replication by other mechanisms besides A3G degradation is a matter of debate [45]. 

A detailed knowledge of the protein domains involved in this interaction is extremely important for the rational design of new antiviral drugs. To this date, several regions in Vif and A3G proteins have been mapped, and the effect on their interaction has been studied. Nevertheless, the three-dimensional molecular structure of both proteins was not yet determined, and only their structure homology modelling was reported [82, 83].

As described above, the most important domains of A3G responsible for Vif interaction involve the asparagine at position 128 and its surrounding residues. The charge of D128 amino acid in A3G markedly influences the interaction with Vif indicating that the protein binding is dependent on electrostatic forces [68]. Thus, chemical compounds targeting this region could be effective in preventing Vif-A3G interaction. Importantly, molecules targeting amino acid 128 are not predicted to interfere with A3G enzymatic activity which is conferred by other domains of the protein [107, 108]. 

In addition to this region, other motifs in A3G have been validated as potential targets for antiviral drugs. As previously discussed, other domains of A3G are involved in the mechanism of Vif binding. This conclusion probably reflects a broad structural interface that stabilizes Vif-A3G interaction. However, it is not known yet if these regions converge towards a unique functional structure since we lack a high-resolution structure of A3G. Therefore, the development of new drugs targeting D128 residue should take into account the involvement of multiple adjacent regions.

The Vif protein is also a potential target for HIV-1 drug therapy. In contrast to A3G, Vif has a scattered localization of motifs capable to mediate its interaction with APOBEC3 proteins. Thus, it is conceivable that the mechanism by which Vif recognizes A3G may involve multiple and conserved functional structures in the viral protein. Whether these structures interact alone or in synergy towards A3G binding is not yet known. 

The N-terminal region of Vif has been implicated in binding to A3G, whereas the protein motifs that mediate interaction with Cul5-E3 ligase complex and promote A3G degradation are concentrated in the C-terminal region of Vif [58, 67–70]. Specifically, in the context of the ^144^SLQXLA^149^ motif, lysine at position 145 is crucial for binding to EloC and to initiate the process of A3G-targeted degradation. Other regions, like the zinc-binding HCCH motif, and the Vif multimerization domain (^161^PPLP^164^) are also present in the C-terminus of Vif and may constitute alternative inhibition motifs. It has been shown that mutations in the ^161^PPLP^164^ motif, reduced Vif binding and degradation of A3G without affecting the interaction of Vif with Elongin C and Cullin5 [100]. Interestingly, when an anti-viral peptide that mimics the Vif PPLP dimerization domain was used, the amount of A3G incorporation into wild-type HIV-1 particles increased [109]. Thus, small molecules that target this motif could be developed as antiviral drugs to block the Vif-mediated inhibition of A3G and A3F activity.

Although the three-dimensional structure of HIV-1 Vif was not yet determined, a close perspective has been developed by comparative modelling [109]. Using molecular dynamics simulation, it was shown that mutations of critical residues led to the disruption of Vif and EloB-EloC interaction, consistent with experimental observations. These novel homology models of Vif can therefore provide structural information for investigating critical domains for protein neutralization. 

While a complete and accurate structure is not available, antiviral drugs that could inhibit Vif and enhance A3G/A3F activity are emerging as attractive candidates [110–113]. For example, a small Vif antagonist that increases the intracellular level of A3G and its incorporation into virions in a Vif-dependent manner has been identified [114]. This compound was shown to enhance the degradation of Vif in an A3G-dependent manner without being a general inhibitor of the proteasome-mediated protein degradation [114]. 

Nonetheless, developing specific and effective small chemical inhibitors to directly inhibit Vif-A3G interaction faces many challenges due to the multiple binding regions involved. In addition, *in vitro* binding assays and cell-based assays that have been used to decipher the dynamic principles behind protein functional association make it sometimes difficult to assess the *in vivo* significance of the results. In particular, co-immunoprecipitation assays that have been commonly used to study specific domains involved in Vif-A3G interaction are questioned. One study has shown that Vif was able to inhibit virion encapsidation and the antiviral activity of an A3G degradation resistant mutant (C97A) [115], suggesting a direct inhibition of A3G by Vif. However, the authors could not rule out the possibility that Vif-A3G complexes could have been formed after cell lysis during co-immunoprecipitation assays. Finally, no one so far has been able to demonstrate a direct Vif-A3G interaction with purified components at physiological concentrations. Therefore, we cannot rule out the need for post-translational modifications or interactions during synthesis, or the need for additional components in the interaction complex. 

It is plausible that the binding features of Vif-EloC interaction present mechanistic specificities that would be optimal for the rational development of a Vif antagonist. Moreover, the specific and stable interaction of ^144^SLQXLA^149^ domain of Vif with EloC will probably result in a better approach for drug screening. 

However, attention must be taken when designing new antiviral drugs since incomplete Vif inhibition could result in increased A3G levels in the cell enough to exert an intermediate level of mutational pressure on the HIV-1 genome resulting in a “sub-error catastrophe.” This could accelerate viral evolution instead of inducing a population collapse resulting in anti-retroviral resistance [116] and immune escape. Despite some controversy remaining on that subject, a therapeutic strategy that could amplify this nonmutagenic phenotype without enhancing cytidine deamination may be an alternative to suppress viral replication. In addition, A3G and A3F may also function through other mechanisms that do not necessarily require deamination [8, 117, 118].

In conclusion, antiviral drugs that could inhibit Vif and enhance A3G/A3F activity are emerging as attractive candidates [110–113]. Nevertheless, the potential outcome of a Vif-based intervention must be examined rigorously both *in vitro* and *in vivo* prior to clinical deployment.
